# A Miniaturized Colorimeter with a Novel Design and High Precision for Photometric Detection

**DOI:** 10.3390/s18030818

**Published:** 2018-02-14

**Authors:** Jun-Chao Yan, Yan Chen, Yu Pang, Jan Slavik, Yun-Fei Zhao, Xiao-Ming Wu, Yi Yang, Si-Fan Yang, Tian-Ling Ren

**Affiliations:** 1 Institute of Microelectronics, Tsinghua University, Beijing 100084, China; yanjc15@mails.tsinghua.edu.cn (J.-C.Y.); ychen2011@tsinghua.edu.cn (Y.C.); pangyu.1990@163.com (Y.P.); Jan.slavik@ceitec.vutbr.cz (J.S.); zhao90yun01fei31@sina.com (Y.-F.Z.); imewuxm@tsinghua.edu.cn (X.-M.W.); yiyang@tsinghua.edu.cn (Y.Y.); fyang@sz.tsinghua.edu.cn (S.-F.Y.); 2 Tsinghua National Laboratory for Information Science and Technology (TNList), Tsinghua University, Beijing 100084, China; 3 Graduate School at Shenzhen, Tsinghua University, Shenzhen 518055, China; 4 Department of Electronic Engineering, Tsinghua University, Beijing 100084, China; 5 Central Institute of Technology (CEITEC), Brno University of Technology, Purkynova 123, 61200 Brno, Czech Republic

**Keywords:** colorimeter, phosphorus detection, Beer-Lambert law, microflow cell, photodetection sensor

## Abstract

Water quality detection plays an increasingly important role in environmental protection. In this work, a novel colorimeter based on the Beer-Lambert law was designed for chemical element detection in water with high precision and miniaturized structure. As an example, the colorimeter can detect phosphorus, which was accomplished in this article to evaluate the performance. Simultaneously, a modified algorithm was applied to extend the linear measurable range. The colorimeter encompassed a near infrared laser source, a microflow cell based on microfluidic technology and a light-sensitive detector, then Micro-Electro-Mechanical System (MEMS) processing technology was used to form a stable integrated structure. Experiments were performed based on the ammonium molybdate spectrophotometric method, including the preparation of phosphorus standard solution, reducing agent, chromogenic agent and color reaction. The device can obtain a wide linear response range (0.05 mg/L up to 7.60 mg/L), a wide reliable measuring range up to 10.16 mg/L after using a novel algorithm, and a low limit of detection (0.02 mg/L). The size of flow cell in this design is 18 mm × 2.0 mm × 800 μm, obtaining a low reagent consumption of 0.004 mg ascorbic acid and 0.011 mg ammonium molybdate per determination. Achieving these advantages of miniaturized volume, high precision and low cost, the design can also be used in automated in situ detection.

## 1. Introduction

As we all know, water is an important natural resource for the survival and development of human beings. With increasing water consumption and water pollution, more and more countries face the problem of water shortage, which seriously restricts the development of the economy and society [1,2,3,4,5]. Therefore, water quality monitoring is attracting increasing attention. Currently, the monitoring instruments are bulky, expensive and time-consuming. Miniaturization of design can improve the flexibility and automation of water quality monitoring [6,7,8]. Pogrebniak et al. prepared a multi-commutated flow analysis system for phosphate monitoring based on fluorescence microdetectors [9]. They used two dedicated detectors made of three integrated light emitting diodes each and operating according to the fluorometric paired emitter detector diode principle. However, no efforts in miniaturization and integration were made. Christine Jeyaseelan used differential pulse polarography to trace determination of dexamethasone sodium phosphate [10], while not trying to achieve a miniaturized buffer system. Yang and Pagliano employed the ion exclusion chromatography method for direct determination of dissolved phosphate [11]; however, the experimental equipment used in this method is so large and expensive that it cannot be used in field automation testing. Shyla and Mahadevaiah adopted a spectrophotometric method for the determination of phosphate in soil, detergents and water [12], whereas the spectrophotometric method using a spectrophotometer is usually employed in laboratory environment. Meanwhile, commercial detectors based on colorimetry have been widely used in some analysis fields. Some researchers summarized the detection methods and compared their performance [7,13]. However, most methods have the limitation of having large-volume sensors, high cost, and complex chemical and experimental conditions. The availability of a simple and compact device will enable rapid detection and immediate assessment. In this work, we designed a colorimeter with a novel structure based on the Beer-Lambert law and MEMS (Micro-Electro-Mechanical System) technology to obtain a miniaturized photometric detection with high precision, while a modified algorithm is applied to extend the linear measuring range.

The Beer-Lambert law reflects the attenuation of light due to absorption of different materials and optical paths in the solution where the light is traveling [14,15]. It has been widely used in many fields such as optical calibration, chemical analysis measurements, biological information detection and so on [16,17,18,19]. By analyzing the reflectance and transmittance spectroscopy, substances with a specific absorption wavelength can be observed [20,21,22]. According to the absorption spectrum of phosphorus, the samples used in this work have an absorption peak at 880 nm; therefore, we chose a near-infrared laser that emits at the wavelength of 880 nm [22,23,24]. A photodiode was used as the detector of light intensity, which had a linear response to light intensity [25,26]. The flow cell was fabricated using microfluidic technology, and was assembled to an integrated system [27,28,29,30,31].

In order to test the performance, standard solution with different phosphorus concentrations were prepared, while ascorbic acid was used as the reducing agent and ammonium molybdate solution was used as the chromogenic reagent. Then phosphorus was reduced by ascorbic acid, and a blue complex was formed by the color reaction of ammonium molybdate solution [24,32,33], which absorbed the maximum intensity at a wavelength of 880 nm. Assays were implemented by employing a digital source meter as data acquisition system; software was developed as the visual display of data. A solenoid mini-pump was used for fluid propelling [34,35,36,37,38]. In this work, the effect of residual measurement was considered to extend the linear response range, which is superior to other methods in phosphorus detection [39,40,41,42]. The effect of interferences is determined in order to demonstrate the selectivity of our device. Compared with commercial colorimeters, the volume of our device can be miniaturized because of microfluidic technology. Furthermore, the low cost, less consumption of samples, wider range of applicability and high precision may extend this device for fieldwork water monitoring.

## 2. Materials and Methods

### 2.1. Theoretical Principle

The basic principle of our sensor is the Beer-Lambert law, which is a traditional attenuation theory for light transfer and can be applied to detect the ion concentrations in liquid. It exhibits a linear relationship between the analyte and light attenuation. The Beer-Lambert law is given by:

(1) $$A=-\log_{10}\frac{I_{t}}{I_{0}}=\log_{10}\frac{1}{T}=klc,$$

where A is the absorbance of the sample solution, *I*_0_ is the initial light intensity before passing through solution, *I_t_* is the intensity of transmitted light, *T* is the transmittance, *k* is the attenuation coefficient, *l* is the optical path length crossing over the sample solution and *c* is the concentration of the substance to be measured [14].

**Figure 1:**
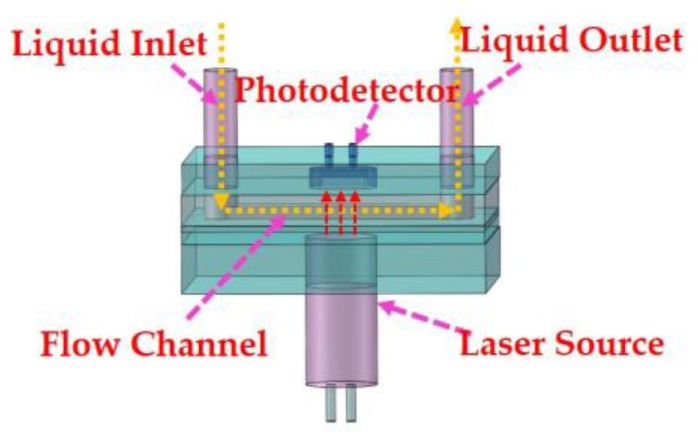
The structure design of the colorimeter.

In our design, we applied MEMS (Micro-Electro-Mechanical System) technology to fabricate the device. The MEMS technology enabled the reduction of the length of *l*, which is beneficial to the volume reduction of the sensor. To ensure high precision in the preparation process, a laser source with a specific wavelength was adopted to enhance the value of *k*. The designed structure is shown in Figure 1.

### 2.2. Design of the Colorimeter

#### 2.2.1. Light Source and Photodetector

**Figure 2:**
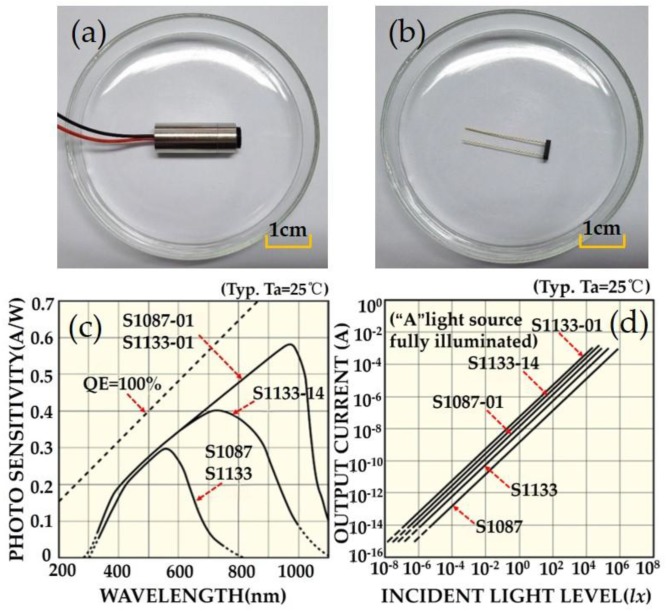
The photograph of (**a**) laser light source and (**b**) photodetector. (**c**) The spectral response curves for different photodetectors, and (**d**) corresponding current response to the light intensity.

In this work, a laser light source with the wavelength of 880 nm was used according to the absorption spectrum of chromogenic reaction. The laser light source works at a power of 5 mW, which can greatly reduce the noise interference caused by power fluctuations [23,43]. The photographs of the light source and the photodetector are shown in Figure 2a,b. The S1133-01 photodetector has a photo-sensitivity of 0.51 A/W at a wavelength of 880 nm, which exhibits the highest sensitivity, see Figure 2c. Figure 2d shows a good linear relationship between the incident light intensity and the output current.

#### 2.2.2. Fabrication of Microflow Cell

**Figure 3:**
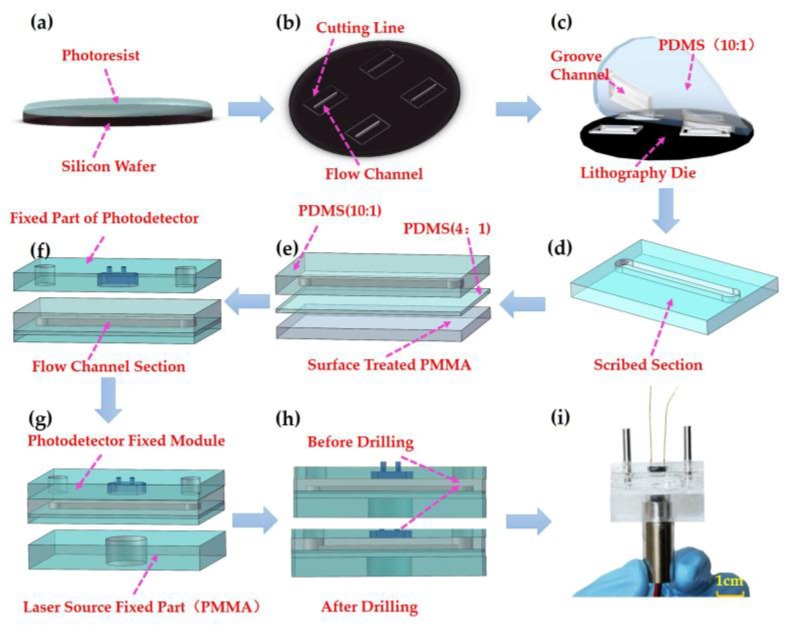
The fabrication process of the device. (**a**) Coating on the wafer. (**b**) Graphic mold after exposure and development process. (**c**) Using polydimethylsiloxane (PDMS) to mold the flow channel. (**d**) Dicing a complete model according to the designed line frames. (**e**) Bonding the flow channel with PDMS at the ratio of 4:1, using the polymethyl methacrylate (PMMA) to increase the stability of the device. (**f**,**g**) Bonding the flow channel with detector and light source module designed previously. (**h**) Punching to get through the flow channel. (**i**) Photograph after the integrated design.

In order to achieve a volume decrease, we adopted the microfluidic technology to fabricate the microflow cell as shown in Figure 1. The soft lithography technology was applied in the fabrication process of the flow cell. Firstly, a mask was prepared using the photographic film. A specific width of flow cell was designed according to the light receiving area of the detector. Then lithography was used to fabricate the micro-channel mold [27,31], as shown in Figure 3a,b.

Polydimethylsiloxane was used to mold the flow channel cell [31,44]. The PDMS and curing agent were mixed with a ratio of 10:1 and 4:1 for different layers in the structure, then was vacuumized to remove bubbles. It was cured at a temperature of 85 °C for 20 min. Then, the mixture of PDMS and curing agent with a ratio of 4:1 was covered on previous substrate bonding layer.

To increase the stability of the device, a thin polymethyl methacrylate (PMMA) layer was adopted as the bonding part with the PDMS layer. Then the sample was cured at a temperature of 80 °C for 1 h. The main procedures of the microchannel cell fabrication process are shown in Figure 3c–e.

#### 2.2.3. Integrated Design of the Colorimeter

To get an integrated system, we used PMMA to fix the light source and detector. For the detector module, two holes were designed at the end of the channel as the inlet and outlet flow channel. After surface modification, the channel and detector modules were bonded. Using drilling technology, we could get through the hole between the detector and microflow channel. Under compression of external force, the device was bonded together using PDMS at a low temperature of 40 °C for 24 h, which belongs to working temperature range for light source and photodetector.

### 2.3. Equipment and Materials

The sensor was measured using the following instruments. A 5 V DC power supply (ITECH IT6953A, Itech Electronics, Nanjing, China) was used for the light source. A biasing voltage of 3 V was applied to the photodetector. A KEITHLEY 2636B SYSTEM source meter was used to collect the output current of the detector. A LabVIEW software was developed to control the digital source meter for data acquisition and visualization display. The home-made colorimetric sensor was described in the previous section. A microfluidic peristaltic pump was used to inject the solution into the fabricated sensor.

### 2.4. Preparation of Reagent Solution

All chemicals were of analytical grade. Ascorbic acid solution was prepared by the following steps: 10 g ascorbic acid was dissolved in 100 mL deionized (DI) water. Ammonium molybdate solution was prepared: the sulfuric acid and DI water were mixed with a ratio of 1:1 (solution 1), 13 g ammonium molybdate was dissolved in 100 mL DI water (solution 2), 0.35 g potassium antimony tartrate was dissolved in DI Water (solution 3). Then the solution 2 was added to 300 mL solution 1, solution 3 was added into prepared mixture. Then Phosphorus Standard Solution was prepared: 0.2191 g KH_2_PO_4_ was dried at temperature of 110 °C for 2 h, and was added to 800 mL DI water, then 5 mL solution 1 was added. Diluted the solution with DI water to 1000 mL. The Phosphorus Standard Solution was prepared by diluting the solution of 50 mg/L with DI water. In our experiment, different concentrations of phosphorus solutions were obtained by diluting the Phosphorus Standard Solution with different ratios of DI water. To verify the selectivity of this sensor, we also prepared Phosphorus Standard Solutions of 4 mg/L with different interference concentrations using the National Standard Sample Solutions. All the solutions were stored at room temperature.

### 2.5. Chromogenic Reaction and Experimental Section

The testing mainly includes the preparation of different phosphorus solutions with different concentrations using Phosphorus Standard Solution, the measurement of samples’ concentrations and data acquisition.

Standard solutions with concentrations ranging from 0.01 mg/L to 15 mg/L were prepared by diluting the 50 mg/L Phosphorus Standard Solution. These solutions were used to determine the current response of the sensor. 10 mL of each solution above was put into the test tube. Then 0.4 mL ascorbic acid solution was added to each of them and mixed well. After 30 s, each solution was mixed with 0.8 mL ammonium molybdate solution. Then stable blue clathrate can be obtained after standing for 15 min.

**Figure 4:**
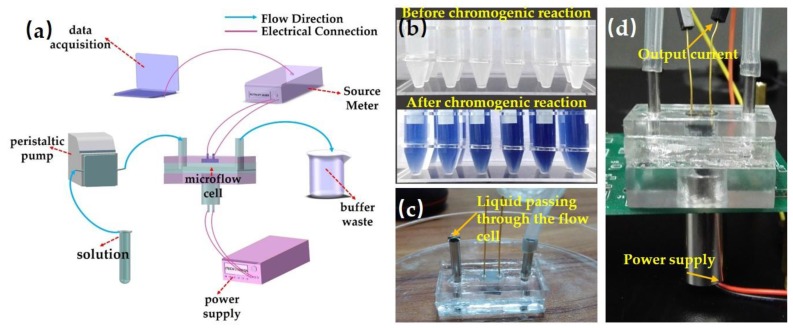
(**a**) The schematics of the experimental setup for photometric detection. (**b**) Solutions to be tested after chromogenic reaction. (**c**) The circulating validation of micro flow cell. (**d**) shows the inlet connected with a peristaltic pump and tested with applied voltages.

The experimental instruments are shown in Figure 4a. Solutions after chromogenic reaction were injected into the microchannel by a peristaltic pump. A computer terminal with developed LabVIEW software was applied for data acquisition. Figure 4b are the solutions to be tested after color reaction. As shown in Figure 4c, the liquid can successfully flow through the microflow cell. In the measurement, the entrance was connected with a peristaltic pump; Figure 4d shows the detailed wire connections of microsystem.

### 2.6. Experimental Procedure

The speed of the peristaltic pump was set up to 10 rpm. The dark current of the photodetector and current signal of DI water (0 mg/L) was recorded before the testing. After testing all the phosphorus solutions, we prepared and tested another three kinds of solution with concentrations of 45, 47.5, 50 mg/L. We could obtain a current of *I_m_* which was available for reducing the effect of residual value and extending the linear range.

In order to verify the consistency of the testing data, an additional experiment was performed to test four representative solutions with concentrations of 0.30, 0.70, 1.20, and 2.55 mg/L. The test involved different concentration-changing sequences, ranging from a low concentration to a high one (0.3, 0.7, 1.2, and 2.55 mg/L), then from a high concentration to a low one (2.55–0.7 mg/L). In addition, the solutions of 0.7, 1.2, and 3.55 mg/L were prepared that would be tested at several times to verify the consistency of the data.

To study the influence of interference elements, we prepared Phosphorus Standard Solutions of 4 mg/L with different concentrations of barium, lead, silver, iron, arsenate, magnesium, chorine, kalium and sulfur, which were also usually found in water. The effect of interferences was determined by testing and analyzing the absorbance differences. We also verified the performance of this sensor for the estimation of phosphorus in seawater.

In this work, many experiments were done to determine the repeatability. Moreover, a set of water samples with unknown concentrations were also prepared, which determines the acquired data availability. In order to verify the data accuracy, the samples were simultaneously analyzed by employing ammonium molybdate spectrophotometric method [26].

## 3. Results and Discussion

### 3.1. Determination of Linear Response Range

**Figure 5:**
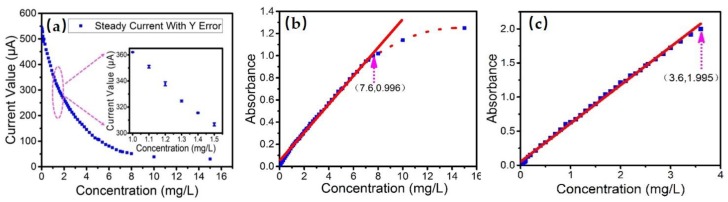
The results of test data statistics and analysis. (**a**) The exponential relation of I-c between the output current and the corresponding sample concentrations. (**b**) The relationship between the obtained absorbance and concentration. (**c**) A linear relationship between absorbance and concentration of reference.

The assays were carried out in a wide range of concentration measurements. Small concentration intervals were tested to determinate the sensitivity in the low-concentration range of 0–0.2 mg/L. We designed plenty of sampling points in a wide range of 0.2 mg/L to 15 mg/L. The points were used to determine the linear response range, while to analyze and reduce the influence of other experimental factors such as the variation of ambient light intensity and the relative error of solution concentrations. The results of assays are shown in Figure 5a, which is consistent with the Beer-Lambert law.

In this experiment, absorbance was calculated using a relationship based on the reference measurement and accomplished with the flow channel filled with carrier solution or DI water. The DI water (0 mg/L) was used as a reference, from which we could obtain a current value I_d_. The output current of the photodetector represents the light intensity. Therefore, a linear relationship exists between the two parameters according to the Beer-Lambert law. The results of these assays can be shown in Figure 5b. For the concentration of 7.6 mg/L, an absorbance of 0.996 is obtained. An obvious deviation from the Beer-Lambert law appears for concentrations higher than 8 mg/L, demonstrating that the law should be used in low-concentration conditions. The reason for the deviation at high concentration is that the premise of the law is to ignore the influence of solution physical properties, such as thermal motion between molecules. The Beer-Lambert law assumes that there is no interaction between solute molecules, which is established in dilute solutions [14,15]. For this experiment, the linear response range is up to 7.6 mg/L. It is a wide measuring range that can be used in most situations, including detection in drinking water, rivers, lakes, etc. [2,7,8,45,46].

The solution was also tested by a spectrophotometer, which was a standard instrument for phosphorus detection with high accuracy [26]. The obtained results are shown in Figure 5c, indicating a linear response range from 0 to 3.6 mg/L. The linear range is less than the scope of our sensor, showing that it can be used for fewer situations compared with our device.

### 3.2. Sensitivity and Detection Limit

**Figure 6:**
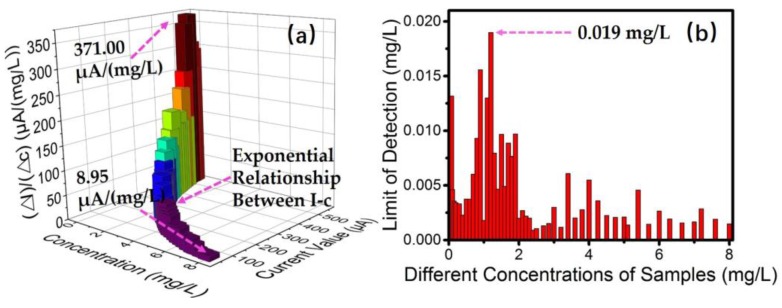
(**a**) A three-dimensional diagram showing the relationship between the output current and the concentration, and the relationship between the sensitivity and the concentration, both of which are exponential. (**b**) The detection limits calculated under different concentrations. The maximum value is 0.019 mg/L considering the worst condition in the point of 1.2 mg/L.

Steady current was obtained for every measurement in the assays, and the current deviation was less than 1.00 μA. Using the solution of 0 mg/L as reference, we can get different current signals for different concentrations of tested solutions. Figure 6a shows the variation of sensitivity in the concentration range of 0–8 mg/L, which shows an exponential change between *r* = (Δ*I*)/(Δ*c*) and concentration. The lowest sensitivity value in the range is 8.95 μA/(mg/L). For the linear relationship of absorbance versus concentration, the sensitivity is 0.1331/(mg/L).

The detection limit, defined as the concentration of the sample when the generated signal is three times of the baseline noise standard deviation, can be expressed as the following equation [47,48,49,50,51]:

(2) D=3N*Q/I,.

where: *D* is the detection limit, *N* is the noise, *Q* is the concentration of sample, and *I* is the generated current signal. *I*/*N* is the signal-to-noise ratio at the concentration of *Q*. Figure 6b reveals the detection limits obtained in all testing. The maximum value of *D* is 0.019 mg/L at the concentration of 1.2 mg/L where the noise is at its maximum, which indicates that the detection limit of the sensor can be defined as 0.02 mg/L.

The result also shows a high resolution of up to 0.01 mg/L for the concentrations range of 1.2 to 8 mg/L. It shows a good performance of lower detection limit and higher accuracy compared with other similar designs for phosphorus detection [7,8,13,47].

### 3.3. Data Consistency Analysis

**Figure 7:**
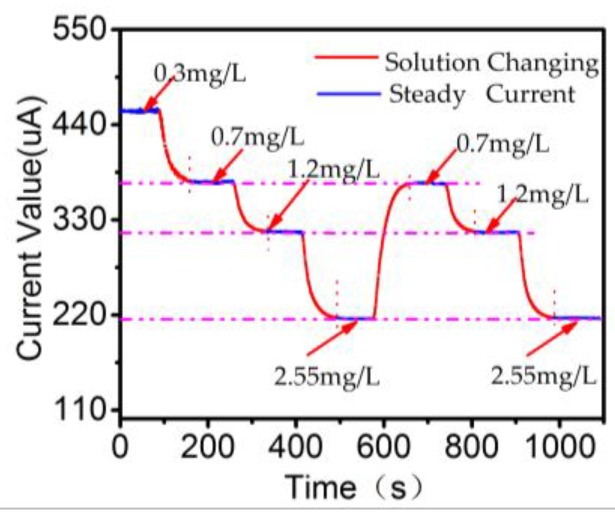
Data recording and analysis of different test process. The current value changes to a steady value for different solution exchange.

In order to verify the consistency of the measured data, we designed four testing solutions including 0.3, 0.7, 1.2, and 2.55 mg/L. Figure 7 shows the result of this experiment. The response time interval of around 50 s between different concentrations reflects the speed of solution-changing process and liquid velocity. The output current for the same concentration after different processes shows the same value.

### 3.4. Study of Interferences

To determine the effect of interference elements, we tested several chemicals that were commonly found as contaminants in water. Different concentrations of interference existed in the solutions with a same phosphorus concentration of 4 mg/L. When the concentration of the interfering ion is 25 times that of the phosphorus, and the variation of absorbance is within the range of ±5%, it can be considered that the interfering ion has no effect on the determination [52]. We firstly prepared and tested the absorbance of solutions with the interfering concentrations of 0 mg/L and 100 mg/L. The results are summarized in Table 1, showing that only arsenate has a significant interference of phosphorus determination. 

To further determine the effect of arsenates, we tested the absorbance of arsenate with arsenate concentration of 0–5.2 mg/L, as shown in Table 2. A significant interference occurred at the arsenate concentration of 3.9 mg/L, which was far higher than that usually found in fresh water. We can also eliminate its effect by using sodium thiosulfate [24].

**Figure 8:**
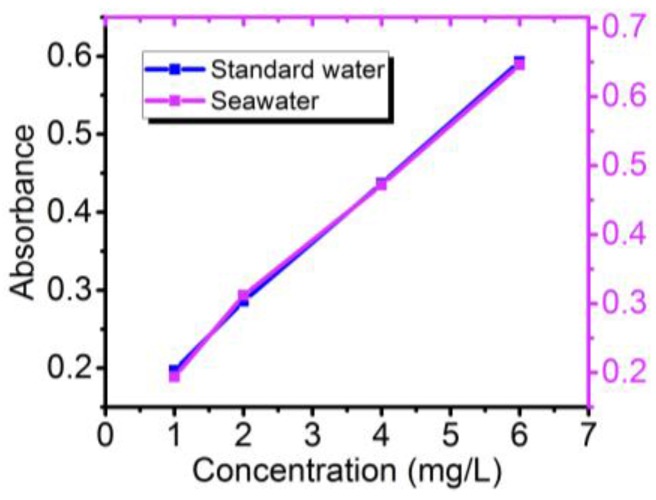
The results of phosphorus determination in standard water and seawater.

Aiming to verify the performance of our sensor for the estimation of phosphorus in seawater, we prepared phosphorus solutions with different concentrations, using the country-level standard seawater (GBW13150) with a salinity value of 3.5%. The result of absorbance measurement is revealed in Figure 8, yielding a linear relationship between absorbance and concentration, which shows that this method is feasible for the estimation of phosphorus in seawater.

### 3.5. Sample Processing

**Figure 9:**
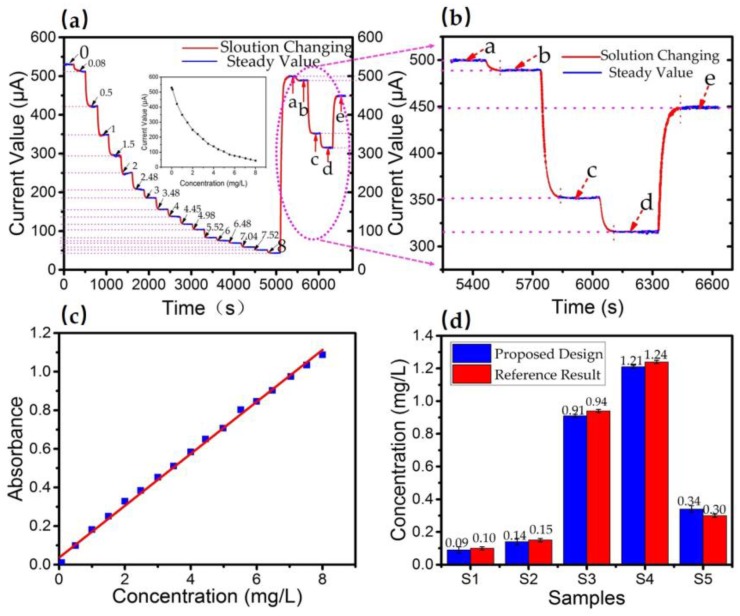
(**a**) A complete record of tracking analysis. From left records refer to blank measurements followed of phosphorus standard solution with concentration ranging from 0.00 to 8.00 mg/L, then followed of five river water samples (a, b, c, d, e). (**b**) Data record for five sample solutions. (**c**) The linear relationship between absorbance and concentration of standard solutions in the assay. (**d**) The concentrations of a, b, c, d, e found by our design and analytical method in laboratory.

While maintaining the working conditions, a set of standard phosphorus solutions and a set of river samples were processed simultaneously to prove the feasibility of our design, as shown in Figure 9a,b. As we can see, the result shows a clear response current value for different concentrations. Figure 9c shows a linear relation between the absorbance and concentrations from absorption analysis. Moreover, samples with unknown concentrations were also analyzed by employing independent procedures. Figure 9d shows the result of determined concentrations compared with reference results. Applying the paired *t*-test, the analysis indicates that there is no significant difference between results of our sensor and reference procedures.

### 3.6. Effect of the Residual Measurement

The current signal generated by the photodetector is affected by illumination of light source through the solution and the scattered light. A fraction of the radiation emitted by the light source may propagate through the wall of the microflow channel, instead of passing through the solution in the microflow cell, before arriving at the photodetector, thus causing a distortion in the measurements (residual measurement). It has been theoretically proved that better adherence to the Beer-Lambert law is achieved considering the stray light measurement and dark measurement for absorbance calculation [53,54]. We have achieved the residual measurement of *I_m_* related to the diffuse radiation in the experiment. Dark measurement *I_k_* is obtained with the light source switched off and the current signal generated using a sample or a standard solution [40,43]. By using the residual measurement as a correction factor, we can obtain the following equation:

(3) $$A=\log_{10}\left[\frac{I_{0}-I_{k}-I_{m}}{I_{t}-I_{k}-I_{m}}\right]=klc,\;$$

where *A* is the absorbance of the sample solution, *I*_0_ is the initial light intensity before passing through solution, *I_t_* is the intensity of transmitted light, *I_k_* is the dark signal of photodiode, which was also generated while the residual measurement was achieved, *I_m_* is the residual measurement we achieved, *k* is the attenuation coefficient, *l* is the optical path length crossing over the sample solution and *c* is the concentration of the absorbing substance.

When measuring the photometer calibration, the stray light measurement was achieved using colored standard solutions, which were obtained using phosphorus solutions with concentrations of 45, 47.5 and 50 mg/L. The signals related to these phosphorus solutions are identical (~21.11 μA). We can suppose that the radiation beam was completely absorbed via propagating through the solution, therefore the detected signal of *I_m_* was generated by the diffuse radiation that arrived at the photodetector.

In this work, we have obtained the *I*_0_ value of 545.38 μA, the small *I_k_* is below 10 pA that is negligible compared with *I*_0_, the result indicates the *I_m_* should be 21.11 μA. We can obtain a 1.27-fold concentration range of 0–10.16 mg/L, which covers larger concentrations.

For other works in phosphorus detection using a light source, the diffuse radiation was not considered and used for the absorbance calculation [6,45,55,56]. The result proves that the modified algorithm could become a tool to extend the linear range of analytical procedures, which provides a novel and practical analysis in photometric detection.

## 4. Conclusions

In this work, we proposed a novel method and design for water quality detection with miniaturization and high precision. The application of MEMS (Micro-Electro-Mechanical System) technology provides a method for further research in miniaturization and integration. The compact size allows the device to be used in portable measurement. The feature of miniaturized flow cell makes it possible to use a low reagent consumption and to produce less waste liquid. The detection limit for phosphorus in water is 0.02 mg/L, with a high resolution of 0.01 mg/L in a large concentration range from 0.05 mg/L to 7.60 mg/L. Compared with other equipment in photometric detection, the effect of residual measurement is considered to extend the linear measurable range, which can be in a range of 0–10.16 mg/L for phosphorus detection, indicating it can be applied in more cases. The advantage of low consumption and small waste liquid makes it easier to be used in in situ detection. The proposed method can also be used to detect other elements, just by changing the light source to the corresponding absorption wavelength. All the above features make it a novel and feasible method for photometric detection in practical applications.

## Figures and Tables

**Table 1:** The analysis of absorbance variance for different interfering chemicals.

Interference	Absorbance at 0 mg/L	Absorbance at 100 mg/L
Barium	0.499	0.512
Lead	0.417	0.408
Silver	0.495	0.519
Iron	0.509	0.530
Arsenate	0.496	0.997
Magnesium	0.468	0.446
Chlorine	0.529	0.551
Kalium	0.489	0.505
Sulfur	0.499	0.505

**Table 2:** The estimation on the interference of arsenate with different concentrations.

**Interference**	0 mg/L	1.3 mg/L	2.6 mg/L	3.9 mg/L	5.2 mg/L
**Arsenate**	0.498	0.507	0.519	0.536	0.561
